# No identifiable Hb1Ac or lifestyle change after a comprehensive diabetes programme including motivational interviewing: A cluster randomised trial

**DOI:** 10.3109/02813432.2013.797178

**Published:** 2013-06

**Authors:** Renate Jansink, Jozé Braspenning, Ellen Keizer, Trudy van der Weijden, Glyn Elwyn, Richard Grol

**Affiliations:** ^1^Scientific Institute for Quality of Healthcare, Radboud University Nijmegen Medical Centre, Nijmegen, the Netherlands; ^2^Department of General Practice, CAPHRI School for Public Health and Primary Care, Maastricht University, Maastricht, the Netherlands; ^3^Department of Primary Care and Public Health, School of Medicine, Cardiff University, Wales, UK

**Keywords:** General practice, lifestyle, primary health care, quality of health care, randomised controlled trial, the Netherlands, type 2 diabetes mellitus

## Abstract

**Objective:**

To study the effectiveness of a comprehensive diabetes programme in general practice that integrates patient-centred lifestyle counselling into structured diabetes care.

**Design and setting:**

Cluster randomised trial in general practices.

**Intervention:**

Nurse-led structured diabetes care with a protocol, record keeping, reminders, and feedback, plus training in motivational interviewing and agenda setting.

**Subjects:**

Primary care nurses in 58 general practices and their 940 type 2 diabetes patients with an HbA1c concentration above 7%, and a body mass index (BMI) above 25 kg/m^2^.

**Main outcome measures:**

HbA1c, diet, and physical activity (medical records and patient questionnaires).

**Results:**

Multilevel linear and logistic regression analyses adjusted for baseline outcomes showed that despite active nurse participation in the intervention, the comprehensive programme was no more effective than usual care after 14 months, as shown by HbA1c levels (difference between groups = 0.13; CI 20.8–0.35) and diet (fat (difference between groups = 0.19; CI 20.82–1.21); vegetables (difference between groups = 0.10; CI-0.21–0.41); fruit (difference between groups = 20.02; CI 20.26–0.22)), and physical activity (difference between groups = 21.15; CI 212.26–9.97), or any of the other measures of clinical parameters, patient's readiness to change, or quality of life.

**Conclusion:**

A comprehensive programme that integrated lifestyle counselling based on motivational interviewing principles integrated into structured diabetes care did not alter HbA1c or the lifestyle related to diet and physical activity. We thus question the impact of motivational interviewing in terms of its ability to improve routine diabetes care in general practice.

The integration of lifestyle counselling based on motivational interviewing (MI) principles into structured diabetes care did not significantly improve diabetes care according to changes:

1. in HbA1c or lifestyle related to diet and physical activity, or any of the other clinical parameters;
2. in the patient's readiness to change or quality of life.

Lifestyle counselling such as MI may be unsuitable for routine diabetes care in general practice.

## Introduction

The prevalence of diabetes is rapidly increasing, due to ageing and changes in lifestyle [1]. The situation is exacerbated by the lack of adherence to the diabetes type 2 recommendations on diet and exercise [2,3]. In many countries, such as the Netherlands, diabetes care has now largely been delegated to primary care nurses. They have to make patients aware of their unhealthy lifestyle and motivate them to change their lifestyle. The complexity of lifestyle change requires a shift from simple advice giving, as described in most diabetes guidelines, to a more patient-centred counselling-based approach [4,5]. Motivational interviewing (MI) has emerged as a promising counselling model for health promotion and disease management, even in brief encounters in general practice [6]. It is a patient-centred method, which makes the patient and professionals jointly responsible for deciding on the treatment plan [7]. Studies have shown that this method can contribute to lifestyle change, such as reducing energy intake from fat, increasing fruit and vegetable consumption [8], increasing physical activity, and lowering weight [9]. Beneficial effects on body mass index, cholesterol, and blood pressure have also been noted [6]. However, most research conducted on this topic has examined the effect of MI on a single behaviour, whereas diabetes is a complex and chronic illness that requires multiple behavioural changes. Compliance with lifestyle advice decreases when several lifestyle behaviours are targeted at the same time [10]. An effective intervention including training nurses in MI will therefore probably not be sufficient, with structured care programmes being needed [11]. Therefore, we developed a comprehensive programme with a focus on training in lifestyle counselling based on MI.

In our study, we assessed the effect of this comprehensive diabetes programme on clinical parameters, lifestyle, patients’ readiness to change lifestyle and quality of life. We also evaluated the participation of nurses in the intervention programme.

## Material and methods

### Study design and study population

General practices were recruited to take part in a cluster randomised trial in the south-eastern part of the Netherlands, from May 2006 to February 2007. Invitation letters were sent with an estimated number of 2500 practices. The 70 practices who volunteered were visited by the first author to explain the study activities. Before randomisation 12 practices withdrew for practical reasons or anticipated disappointment not to be allocated to the intervention group. Randomisation was performed at the level of the general practice (stratified by practice size and level of urbanisation). Patients with type 2 diabetes were eligible to participate when they were younger than 80 years, had an HbA1c above 7%, and a body mass index (BMI) above 25 kg/m^2^. Exclusion criteria were complex comorbidity and treatment in hospital. The research team made a list of all eligible patients by screening the medical files before allocation. Patients were invited by letter and signed an informed consent form.

### Intervention

**Figure 1.**
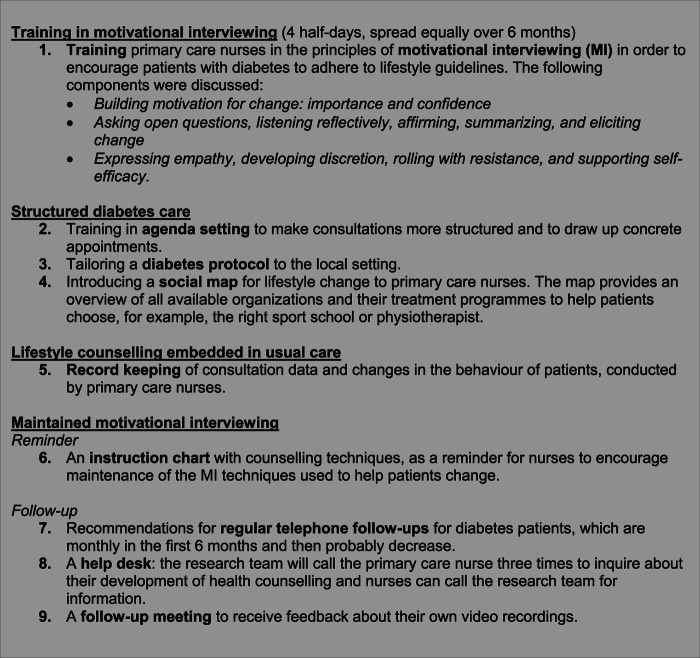
The interventions in the comprehensive programme.

Nurses in the intervention group received a comprehensive programme [12] consisting of (a) training in lifestyle counselling based on motivational interviewing [13]; (b) the introduction of tools for structuring diabetes care, such as training in agenda setting, a local diabetes protocol based on the national guidelines [14] that was discussed with them, and a social map for lifestyle support; (c) instruction on record keeping to integrate lifestyle counselling into general practice; and (d) introduction of tools to sustain improvements including an instruction chart (reminder) [11], regular telephone follow-ups with the target patients, a help desk that also inquired proactively about the progress of diabetes management, and a follow-up meeting for the nurses (Figure 1).

Interventions (a) and (b) took place during the training sessions, which consisted of four half-day training sessions (total 16 hours) spread over the first half year. Nurses attended these sessions in groups of 5–8 outside the practice. The other activities started after these training sessions (after six months) and lasted till the post-measurement after 14 months. The record-keeping and the instruction chart were offered to nurses during the last training session. They received an oral and written explanation of the record-keeping and recommendations for regular telephone follow-ups for diabetes patients. During the intervention period the research team called the practice nurse once a quarter (three times) to enquire about their development of health counselling. The nurses could call the research team for information any time. About four months after the last training session, the nurses were invited to participate in a follow-up meeting to discuss the barriers in practice and to receive feedback about their own video-recording. What went right, and what could be better? The usual care nurses were advised to administer care consistent with current diabetes guidelines.

### Measures and data collection

The primary outcomes were HbA1c and reported changes in lifestyle related to diet and physical activity. Data on diabetes outcome measures (HbA1c, blood pressure, cholesterol, BMI) and process indicators (see Table IV) were extracted from medical records. The research team identified all the eligible patients with an HbA1c of 7% or more. Only data of those patients who had given informed consent were collected by extracting the information manually from the electronic medical records. In case of more measurements per patients within the 12 months’ retrospective window, the most recent value was collected. Patient questionnaires were used to collect data on alcohol, fat, vegetables, and fruit consumption over the past month [15–17]. Physical activity was measured by asking patients to describe a typical week during the last month [18]. Physical activity was also reported more objectively based on a personal activity meter (PAM) and a diary (same week). For each lifestyle aspect the importance of and confidence in changing was rated on a five-point Likert scale; each patient's readiness to change was defined by multiplying the two items [13]. Quality of life was assessed using the Euroqol [19]. Data were gathered at baseline, and after 14 months. In the Dutch diabetes guidelines it is stated that diabetes patients should be seen once a quarter and the HbA1c should be measured yearly. For practical reasons (e.g. holidays, forgotten) it can be difficult to perform the yearly visit. We therefore took a time frame of 14 months.

The exposure of nurses to MI, agenda setting, diabetes protocols, and social maps was measured by recording their attendance at the different training sessions on these subjects. Furthermore, we asked nurses if they used the instruction chart, and we recorded the number of nurses who received three telephone follow-ups from the research team, as well as their participation in the follow-up meeting.

### Sample size

With the intervention used, we expected 50% of the eligible patients to achieve an Hba1c below 7% [12]. Based on an alpha of 5% and a beta of 80% a random sample of 30 general practices with five patients each was needed, taking into account an intra-cluster correlation of 0.05 (patients in practices). For lifestyle we expected a 5% change in usual care, and an extra 10% change due to the intervention [12]. Without changing the other assumptions, a total of 68 practices with 10 patients were needed to detect the expected difference in lifestyle between the intervention and usual care groups. We thus planned to recruit 70 general practices with 700 patients, allowing for some loss.

### Statistical analysis

Means and standard deviations, and percentages where appropriate, were used to summarise the characteristics of the general practices, nurses, and patients. Comparisons between the intervention and usual care arms were adjusted for clustering within practices. Continuous outcome measures (clinical outcomes, reported aspects of lifestyle, and quality of life) were analysed with multilevel linear regression in SPSS. The HbA1c was added as a continuous measure in the statistical model to avoid loss of power. Binary outcome measures (patients’ readiness to change and diabetes process indicators) were analysed using multilevel logistic regression in SAS. In these analyses baseline measures were defined as a separate predictor in the model. We also adjusted the models for baseline characteristics (from practices, nurses, or patients) that differed significantly between the intervention and usual care groups.

## Results

### Study population

**Figure 2.**
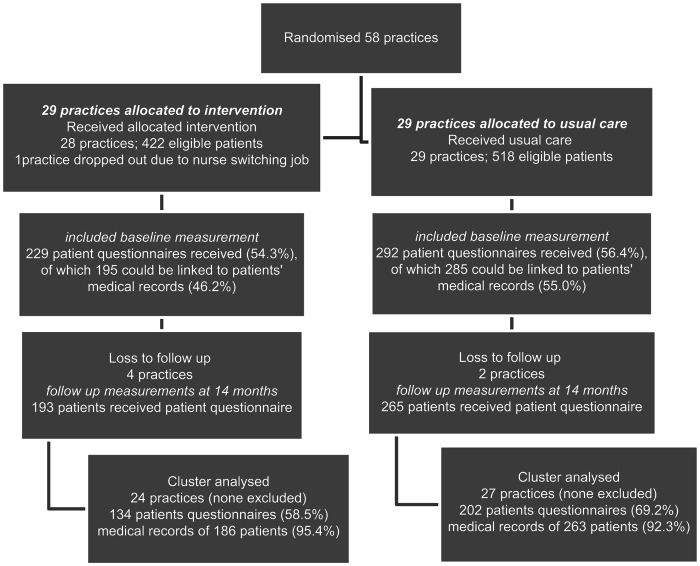
Flow diagram of general practices and patients at different stages (enrolment, allocation, baseline measurement, follow-up, and analysis) of the trial.

Figure 2 presents the number of general practices and participants in this trial. Table I presents the baseline characteristics of the general practices, nurses, patients, and the baseline values regarding outcome measures, lifestyle, and quality of life.

**Table I.** Baseline characteristics of general practices, primary care nurses, patients, and baseline values of measures.

	Intervention		Usual care	
		SD		SD
General practices (n = 53):^a^	25		28	
Mean number of patients in general practice^b^	4566	2703.8	5657	4153.3
Mean number of patients with type 2 diabetes in general practice	195	109.6	202	119.4
Mean FTE^c^ primary care nurses in general practice	0.6	0.3	0.8	0.6
Primary care nurses (n = 53):^d^	25		28	
Mean age in years	40.7	7.8	44.4	6.6
Number of men/number of woman	2/23		1/27	
Mean years of experience with diabetes consultations	3.6	2.1	3.6	2.1
Nurses who were formerly practice assistants	48.0		53.6	
Mean years of experience as practice assistant	4.3	5.3	8.4	8.8
Patients (n = 521):	229		292	
% men	55.9		54.0	
Mean age in years	64.1	8.9	63.9	9.8
Mean duration of diabetes in years	7.5	6.0	7.8	5.8
Baseline (n = 336):	134		202	
*Outcome measures:*				
HbA1c, %	7.8	0.9	7.7	0.7
Systolic blood pressure, mm Hg	144.4	20.3	140.7	18.0
Diastolic blood pressure, mm Hg	81.9	10.6	79.9	9.9
LDL, mmol/l	2.8	1.0	2.5	0.8
Total cholesterol, mmol/l	4.7	1.0	4.5	1.0
BMI, kg/m^2^	30.7	4.2	30.7	4.2
*Lifestyle:*				
Alcohol, units/day^e^	2.3	1.0	2.2	1.1
Fat score, g/day	14.1	4.5	14.5	4.9
Vegetables, tablespoons/day	2.9	1.6	3.0	1.5
Fruit, pieces/day	1.9	1.1	1.8	1.1
Physical activity, minutes/day	64.8	66.1	58.6	45.1
Pam score^f^	19.2	8.5	21.2	8.0
Low activity, minutes/day	73.2	40.3	77.0	37.0
Medium activity, minutes/day	21.1	23.4	23.4	23.8
High activity, minutes/day	0.3	0.8	0.7	3.3
Diary activity, minutes/day	129.9	77.4	153.9	103.4
*Quality of life:*				
VAS score	74.1	16.2	73.1	13.5

Notes: ^a^Four general practices withdrew from the study before the nurse had completed the questionnaire about the baseline characteristics. ^b^SD = standard deviation. ^c^FTE = full time equivalent; ^d^In six practices two nurses were employed. In such cases we calculated the mean of the nurse characteristics, because the mean values were used in follow-up analyses. ^e^Only people who reported alcohol consumption. ^f^Pam score = Personal activity meter score.

### Follow-up analyses

Based on the medical records of the dropouts at baseline we learned that follow-up was somewhat higher among older patients (63.8 vs. 61.4 years), and patients with a lower BMI (30.8 vs. 32.4) and a lower HbA1c (7.8% vs. 8.0%). At follow-up after 14 months we lost very little information from the medical records, but again not all patient questionnaires were returned (see Figure 2). The follow-up was not affected by age or BMI, but the HbA1c of the non-responders was slightly higher (0.2%) than that of responders.

### Diabetes care

Table II shows that compared with usual care the comprehensive programme did not result in statistically significant improvements in the diabetes outcome measures (HbA1c, blood pressure, cholesterol, BMI). However, the small changes in cholesterol outcomes (LDL and total) had a p < 0.10. Post measurement showed that in the intervention group 34.5% reached the HbA1c target value (below 7%) compared with 34.0% in the usual care group (OR = 1.17, 95% CI = 0.74–1.85, p-value = 0.49). The number of people with an HbA1c above 8.5% decreased in the intervention group from 16.5% to 9.8% and in the usual care group from 11.9% to 9.8% (OR = 1.01, 95% CI = 0.42–2.39, p-value = 0.99). The comprehensive diabetes programme was no more effective than usual care in terms of the reported consumption of alcohol, fat, vegetables and fruit, or physical activity. Physical activity showed different outcomes when measured by questionnaire, personal activity meter, or diary, but none of these outcomes differed between intervention and usual care. The intervention did not increase or decrease quality of life compared to usual care.

**Table II.** Effect of comprehensive diabetes programme on diabetes outcome measures, lifestyle, and quality of life after 14 months’ follow-up.^a^

	Intervention			Usual care			Difference between groups^b^	95% CI	p-value
	M	SD	n	M	SD	n			
Outcome measures:									
HbA_1_c, %	7.3	0.7	129	7.4	1.0	197	0.13	−0.08 – 0.35	0.221
Systolic blood pressure, mm Hg	141.5	17.0	120	137.8	15.8	185	−1.98	−5.63 – 1.67	0.279
Diastolic blood pressure, mm Hg	79.5	8.4	120	77.6	9.2	120	−1.17	−3.41 – 1.07	0.294
LDL, mmol/l	2.6	0.8	106	2.4	0.6	178	−0.15	−0,32 – −0.02	0.081
Total cholesterol, mmol/l	4.5	1.0	122	4.2	0.8	186	−0.21	−0.41 – 0.00	0.051
BMI, kg/m^2^	30.2	4.0	106	30.5	4.6	179	0.36	−0.19 – 0.90	0.198
Lifestyle:									
Alcohol, units/day^c^	2.2	1.0	58	2.2	1.1	95	0.04	−0.14 – 0.23	0.647
Fat score, g/day	13.9	5.4	105	14.2	6.1	163	0.19	−0.82 – 1.21	0.708
Vegetables, tablespoons/day	3.1	1.6	102	3.1	1.5	165	0.10	−0.21 – 0.41	0.518
Fruit, pieces/day	1.8	1.1	119	1.7	1.2	173	−0.02	−0.26 – 0.22	0.884
Physical activity, minutes/day	62.8	69.6	124	59.1	51.3	171	−1.15	−12.26 – 9.97	0.839
Pam score^d^									
Low activity, minutes/day	78.3	40.3	78	78.8	39.8	120	−2.70	−10.52 – 5.14	0.498
Medium activity, minutes/day	22.5	27.0	78	22.6	20.2	120	−1.46	−6.80 – 3.89	0.592
High activity, minutes/day	0.4	0.9	78	1.0	6.5	120	0.18	−0.65 – 1.01	0.669
Diary activity, minutes/day	152.9	97.6	84	157.4	89.0	128	−19.36	−39.97 – 1.26	0.066
Quality of life:									
VAS score	75.3	16.2	111	73.5	13.6	171	−1.27	−4.50 1.97	0.441

Notes: ^a^Adjusted for baseline measures, nurses’ years of experience, and cluster effects. ^b^Difference between intervention and usual care group (reference group). ^c^Only people who reported alcohol consumption. ^d^Pam score = Personal activity meter score.

Table III indicates that the intervention did not change any aspect of patients’ readiness to change their lifestyle. The number of participants who themselves reported not meeting the norm was lower than expected in a group with a BMI above 25 kg/m^2^.

**Table III.** Effect of comprehensive diabetes programme on patient's readiness to change lifestyle^a^ after 14 months’ follow-up.^b^

	Intervention			Usual care					
	M	SD	n	M	SD	n	B	95% CI	p-value
Alcohol, units/week^c^	12.8	4.1	11	10.5	3.6	18	−1.03	−3.93 – 1.86	0.471
Fat, g/day	14.8	6.0	30	13.0	3.9	43	0.21	−3.19 – 3.60	0.901
Vegetables, g/day	12.5	5.1	89	13.7	4.5	150	0.74	−0.47 – 1.96	0.228
Fruit, pieces/day	12.5	4.4	71	11.9	4.1	116	−0.38	−1.82 – 1.05	0.597
Physical activity, minutes/day	11.7	5.4	63	11.1	4.1	126	−0.48	−2.13 – 1.17	0.563

Notes: ^a^Only for patients who did not reach the norm for lifestyle; range 1–25. ^b^Adjusted for baseline measures and nurses’ years of experience. ^c^Only people who reported alcohol consumption.

**Table IV.** Effect of comprehensive diabetes programme on diabetes process indicators after 14 months’ follow-up.^a^

	Number of patients (%)				
	Intervention n = 186	Usual care n = 263	Odds ratio	95% CI	p-value
Dietary advice	104 (56)	119 (45)	0.96	0.86 – 1.06	0.838
Physical activity advice	106 (57)	116 (44)	0.96	0.87 – 1.06	0.984
HbA1c checked	182 (99)	251 (95)	2.13	0.60 – 7.53	0.239
Blood pressure checked	185 (100)	245 (93)	13.59	1.79 – 103.37	*0.01*
Total cholesterol checked	174 (94)	242 (92)	0.99	0.94 – 1.04	0.554
LDL cholesterol checked	169 (91)	239 (91)	2.25	1.01 – 5.00	0.838
Creatinine (serum) checked	174 (94)	242 (92)	1.02	0.96 – 1.07	0.322
Microalbuminuria checked	163 (88)	223 (85)	0.98	0.93 – 1.04	0.931
Eye examination^b^	61 (33)	106 (40)	1.01	0.96 – 1.06	0.717
Foot examination	131 (70)	205 (78)	0.98	0.94 – 1.02	0.101
BMI determined	166 (89)	230 (87)	0.97	0.91 – 1.04	0.832
Cholesterol-lowering medication	80 (43)	93 (36)	0.97	0.88 – 1.07	0.955

Notes: ^a^Adjusted for baseline measures and nurses’ years of experience. ^b^In general once in 24 months, but eye examinations were recorded in the last 12 months.

The findings on diabetes process indicators revealed that the number of patients receiving lifestyle advice neither increased nor decreased as a result of the comprehensive diabetes programme (Table IV). The probability of annual checks on HbA1c, blood pressure, and LDL increased, but only significantly for blood pressure. However, the high adherence rates at baseline in both the intervention and usual care group suggested that this significant finding is not clinically relevant.

Nurses in the intervention actively participated in the programme, with 93% attending at least three out of four MI training sessions. The social maps and local diabetes protocols were discussed by 74% of the nurses. All practices received the three quarterly telephone follow-ups from the research team after training. During these conversations, nurses indicated that the instruction chart was very useful. Most of them had the chart on their desk and used it during or after consultations. Although the nurses requested an MI follow-up meeting, participation was low (37%).

## Discussion

### Statement of principal findings

The comprehensive diabetes programme had no effect on HbA1c or reported aspects of lifestyle, nor on the other diabetes outcome measures or quality of life. Patients with type 2 diabetes were no more ready to change lifestyle in the intervention practices than in usual care, and the adherence of nurses to guidelines for process measures showed no relevant improvement, despite their active participation in the training programme.

### Strengths and limitations of the study

Strengths include the cluster RCT design, and the variety of measures (outcome and process, subjective and objective). As 70 out of 2500 practices participated voluntarily, it can be assumed that only practices that were enthusiastic about improving diabetes care by using MI were recruited. This could have affected the study results. However, improvement was not shown in the intervention or in the control group. A limitation of the study is the loss to follow-up in the lifestyle measures from the patient questionnaire. At baseline and follow-up we lost participants with a relatively higher HbA1c than those who remained within the study, which narrowed the room for improvement in HbA1c. We also noticed that the proportion of measurements differed between the intervention and usual care group. Although we formulated strict inclusion criteria and performed randomisation, it is possible that the population in the intervention and usual care group differed on variables beyond our set of measurements. Another limitation was the underpowered nature of the patient questionnaires. However, based on the difference between groups, confidence intervals, and p-values in Table II, we can assume that a larger sample size would not have led to any significant changes either. The follow-up period was 14 months. It is theoretically possible that the effect can only be seen in the long term. But this contradicts other studies that showed an effect of MI directly after its introduction [6,20].

### Comparison with existing literature

The comprehensive diabetes programme was based on elements that had proved to be effective such as structured diabetes management [11] and motivational interviewing [6,8,9]. Previous research has reported that for patients with type 2 diabetes the use of MI can improve glucose control, dietary changes, smoking, weight, physical activity, motivation for lifestyle change, and adherence to diabetes guidelines [6,8,9,21]. Knowing that dietary advice and physical activity are associated with a lower HbA1c [22,23], we would have expected our comprehensive diabetes programme to have an effect on HbA1c and lifestyle. However, recently more studies have questioned the effectiveness of MI in terms of clinical outcomes, lifestyle, quality of life, and self-efficacy for patients with type 1 and 2 diabetes or in cardiovascular risk management in routine care in general practice [24–27].

### Explanation of the findings

There are several possible reasons why our trial failed to demonstrate any effectiveness of the comprehensive diabetes programme. A thought could be that Dutch diabetes care is already on a high level as the guideline was introduced in 1989, which makes further improvement difficult as could be shown by the mean HbA1c of 7.8% at baseline. Improvement on BMI, however, was certainly possible in the study population. Another explanation could be that the education of the nurses was of such level that lifestyle education was performed on almost the same level in both the intervention and control group, and the training programme hardly added value. Nurses in the Netherlands are trained in a three- to four-year curriculum (middle or higher education) and afterwards they can specialise in primary care following a one- or two-year curriculum. Lifestyle counselling is part of the curriculum, but not specifically focused on motivational interviewing. The education is a prerequisite for effective lifestyle counselling, but no guarantee for reaching good outcomes. The lifestyle outcomes in the study showed that there could be improvement. We could consider the quality of our training programme itself, but there are no comparable data. It is only known that the four training sessions offered were sufficient [28]. As nurses themselves asked for an extra session, it can be concluded that more support was desirable. Perhaps training on the job can help to produce more effective lifestyle counselling [29].

As room for improvement was available and nurses in the intervention group were better equipped to perform lifestyle counselling, the study results could be explained by the study design itself. Some of its weaknesses have already been discussed, but there impact seems to be limited. Could it be that the time frame chosen was too short? Fourteen months after the intervention the post measurements took place, but not all of the patients were seen at the same time. An extra analysis on this subject showed that on average the measurements were performed in the midst of the time frame. It can be assumed that lifestyle changes take more time, but earlier studies have found MI effects after 3–4 months [6,30].

Explanations can also be found in our target population. MI was originally developed for substance abuse [13], requiring a single behavioural change, whereas diabetes is a complex chronic illness that requires multiple behavioural changes. MI may be less effective for multiple behavioural changes, despite the fact that our nurses were trained to set the agenda. In case of diabetes it may be better to organise a setting that is explicitly dedicated to MI. Separate MI sessions have been shown to be successful [31]. In our programme the counselling strategy was applied during regular nurse consultations. Or are we being naive? Targeting changes in biomedical parameters and lifestyle in an elderly population with type 2 diabetes is a battle involving a complicated social, psychological, and physiological web of related issues [32]. Our comprehensive diabetes programme may not have been nearly comprehensive enough.

### Implications for future research on clinical practice

Our results indicate a need for further research on lifestyle counselling embedded in primary care and the assessment of factors influencing the use of such counselling strategies for a better understanding of the applicability of interventions in diabetes care. One approach may lie in the argument that the environment in which we live may be the driving force behind many of our less healthful lifestyle habits [33]. A health protection approach with a possible role for the polypill to reduce cardiovascular risk may be more effective than a motivational intervention [34–36]. Another direction can be an investment in acquiring knowledge on *personalised* lifestyle counselling. Is it possible to customise lifestyle counselling based on genetic or other information? In this case MI will be offered to those patients who will benefit most instead of being part of routine diabetes care. Nurses can focus energy into work for this group with potentially more fruitful results.

## Funding

This study was funded by ZonMW – the Netherlands Organization for Health Research and Development, 945-16-113.

## Ethics approval

The medical ethics committee of the University Medical Centre Nijmegen approved the study. Current Controlled Trials ISRCTN68707773.
